# Molecular control of stomatal development

**DOI:** 10.1042/BCJ20170413

**Published:** 2018-01-31

**Authors:** Nicholas Zoulias, Emily L. Harrison, Stuart A. Casson, Julie E. Gray

**Affiliations:** Department of Molecular Biology and Biotechnology, University of Sheffield, Sheffield S10 2TN, U.K.

**Keywords:** guard cell, peptide ligand, plant biology, receptor kinase, transcription factors

## Abstract

Plants have evolved developmental plasticity which allows the up- or down-regulation of photosynthetic and water loss capacities as new leaves emerge. This developmental plasticity enables plants to maximise fitness and to survive under differing environments. Stomata play a pivotal role in this adaptive process. These microscopic pores in the epidermis of leaves control gas exchange between the plant and its surrounding environment. Stomatal development involves regulated cell fate decisions that ensure optimal stomatal density and spacing, enabling efficient gas exchange. The cellular patterning process is regulated by a complex signalling pathway involving extracellular ligand–receptor interactions, which, in turn, modulate the activity of three master transcription factors essential for the formation of stomata. Here, we review the current understanding of the biochemical interactions between the epidermal patterning factor ligands and the ERECTA family of leucine-rich repeat receptor kinases. We discuss how this leads to activation of a kinase cascade, regulation of the bHLH transcription factor SPEECHLESS and its relatives, and ultimately alters stomatal production.

## Introduction

Stomata play a pivotal role in photosynthesis by maintaining the balance of gas exchange between the aerial parts of the plant and the atmosphere [1]. Stomata can form on both leaf surfaces (amphistomatous) or on a singular surface (hypostomatous). Structurally, they consist of microscopic pores in the epidermis of the leaf encompassed by a pair of guard cells. Stomata can open and close to regulate the diffusion of CO_2_ from the atmosphere into the inner photosynthetic tissues of the plant. Opening of the pore is achieved by solute accumulation in the guard cells, resulting in uptake of water into the cell, and an increase in cell turgor, thus widening the stomatal aperture. Closure is essentially a reversal of this process, although relies on different regulatory signalling pathways [2]. While the primary function of stomatal opening appears to be the passive uptake of CO_2_, it also results in water being lost from the leaf by transpiration. Transpiration is necessary to facilitate the transport of water and micronutrients from the roots to the aerial parts of the plant, but must be tightly regulated to prevent excessive water loss and the potential for the plant to become drought stressed [3,4]. Therefore, stomatal aperture control is dynamic, with opening and closing being co-ordinated to maintain optimum leaf CO_2_ and water potential.

Stomatal aperture control is not the only mechanism through which plants can control leaf gas exchange. As sessile organisms, plants need to modify their developmental processes to improve their reproductive ability and survival in response to surrounding environmental cues. This plasticity in their development also allows plants to modulate water loss and photosynthetic characteristics. To achieve high photosynthetic rates while avoiding dehydration, plants exhibit developmental traits such as shade avoidance, root hydrotropism and regulation of stomatal development to ensure that they have sufficient light, water and CO_2_. Many plant species can regulate developmental pathways to modify the number of stomata on newly developing leaves and thereby alter the maximal and minimal rates of gas exchange [1].

**Figure 1.**
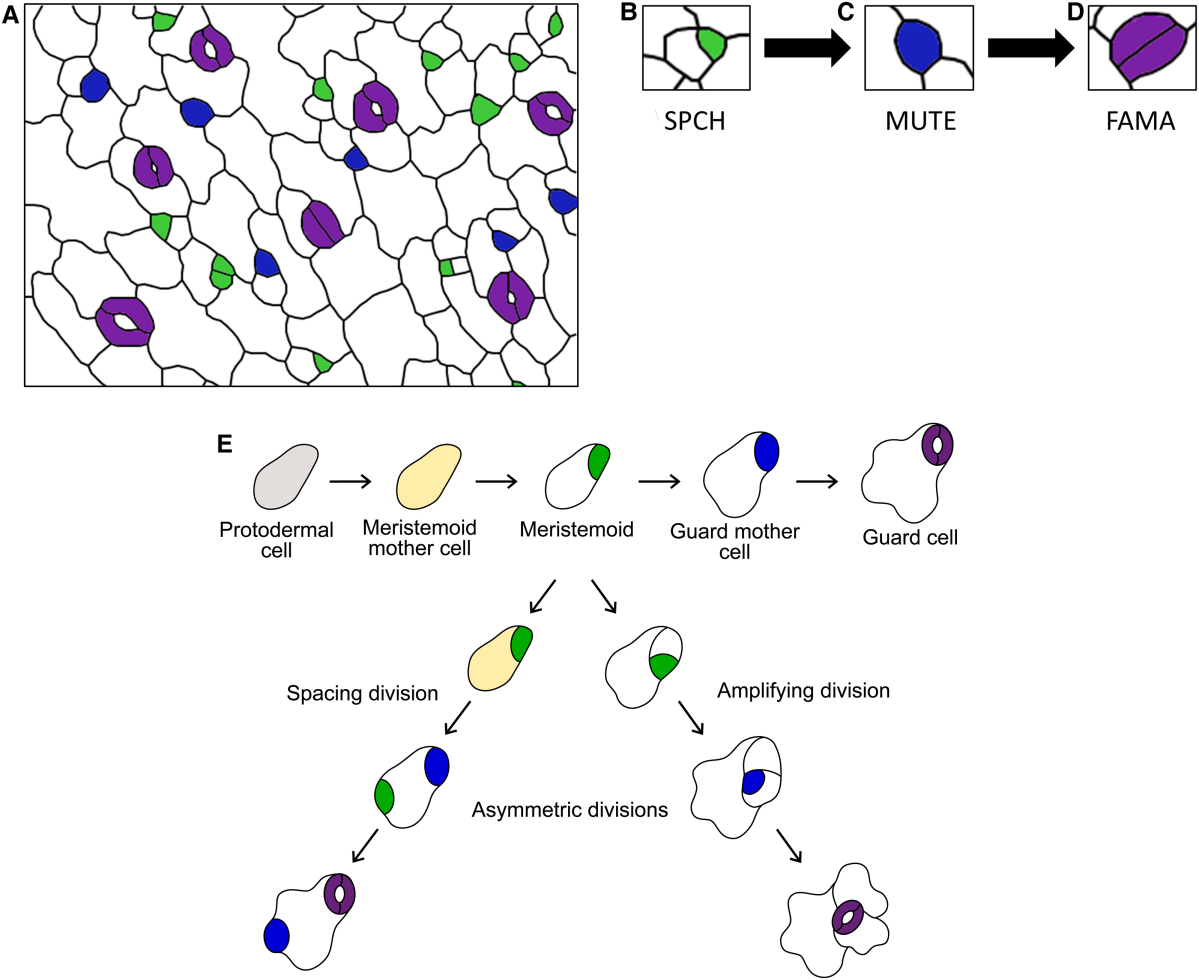
Cells of the stomatal lineage. (**A**) Vectorised confocal image of a young developing *Arabidopsis* abaxial leaf epidermis. This representative epidermis contains cells expressing each of the three bHLH transcription factors that control stomatal development. MMCs and meristemoids which contain SPCH are colored in green (**B**), while GMCs are in blue and contain MUTE (**C**). Newly formed and maturing guard cells are indicated by purple and express FAMA (**D**). Together with the pavement (white) and stomatal lineage ground cells (white), this forms the progression of protodermal cells through the stomatal lineage. (**E**) Cartoon to illustrate the controlled cell divisions and cell fate transitions that regulate stomatal development in the *Arabidopsis* early leaf epidermis.

Stomatal development is a biochemically regulated process in which meristematic protodermal cells undergo a series of asymmetric and symmetric divisions to pattern the leaf epidermis (Figure 1). In this review, we give an overview of the stomatal developmental process before exploring how peptide signals and receptor-like kinases are involved in the regulation of three key transcription factors that control the cellular transitions leading to stomatal formation.

## Stomatal development

The epidermis of young developing leaves consists of undifferentiated protodermal cells, which differentiate into three main cell types in *Arabidopsis thaliana* (*Arabidopsis*): trichomes (or leaf hairs), pavement cells and stomatal guard cells. All three cell types are found on both the upper (adaxial) and lower (abaxial) surfaces of leaves. However, *Arabidopsis* produces many more stomata on the abaxial surface than on the adaxial, most likely to minimise transpirational water loss from the more exposed upper leaf surface [5]. Like trichomes, stomatal precursors develop in a basipetal manner (from the leaf tip to the base), but guard cells are one of the final cell types to differentiate on the leaf epidermis [6,7]. Stomata follow a particular developmental pattern that is known as the one cell spacing rule [8]. This ensures that all stomata are separated by at least one pavement cell which is believed to promote efficient gas exchange while minimising water loss [9].

From protodermal cells, there are several intermediary steps prior to the formation of a mature stoma. This series of fate choices and divisions is known as the stomatal lineage. At many steps along the stomatal developmental pathway, cells may exit from the lineage or have their development arrested; thus, entry into the lineage does not dictate that a cell will ultimately form a stoma [8]. This flexibility allows for the developing leaf to respond to local and systemic signals, as well as the environmental conditions the leaf is experiencing [10,11]. The first step in the lineage is the transition of a protodermal cell to a meristemoid mother cell (MMC), which is then followed by an asymmetric division to form a larger daughter cell [also known as a stomatal lineage ground cell (SLGC)] and a meristemoid (Figure 1) [8]. The meristemoid and larger SLGC pairs have four potential fates: (i) the SLGC can undergo a spacing division to form two meristemoids separated by a pavement cell, (ii) the meristemoid can undergo amplifying divisions to form more SLGCs, (iii) the meristemoid can progress to a guard mother cell (GMC) or (iv) the SLGC and/or meristemoid (rarely) can exit the lineage [8,12,13]. The transition to GMC is a cellular differentiation step which involves the growth and rounding of the cell (Figure 1). The GMC has only two options with regard to differentiation, to divide symmetrically and form a stoma or to arrest development. This complex series of fate changes are guided by master transcription factors essential for the formation of stomata.

**Figure 2.**
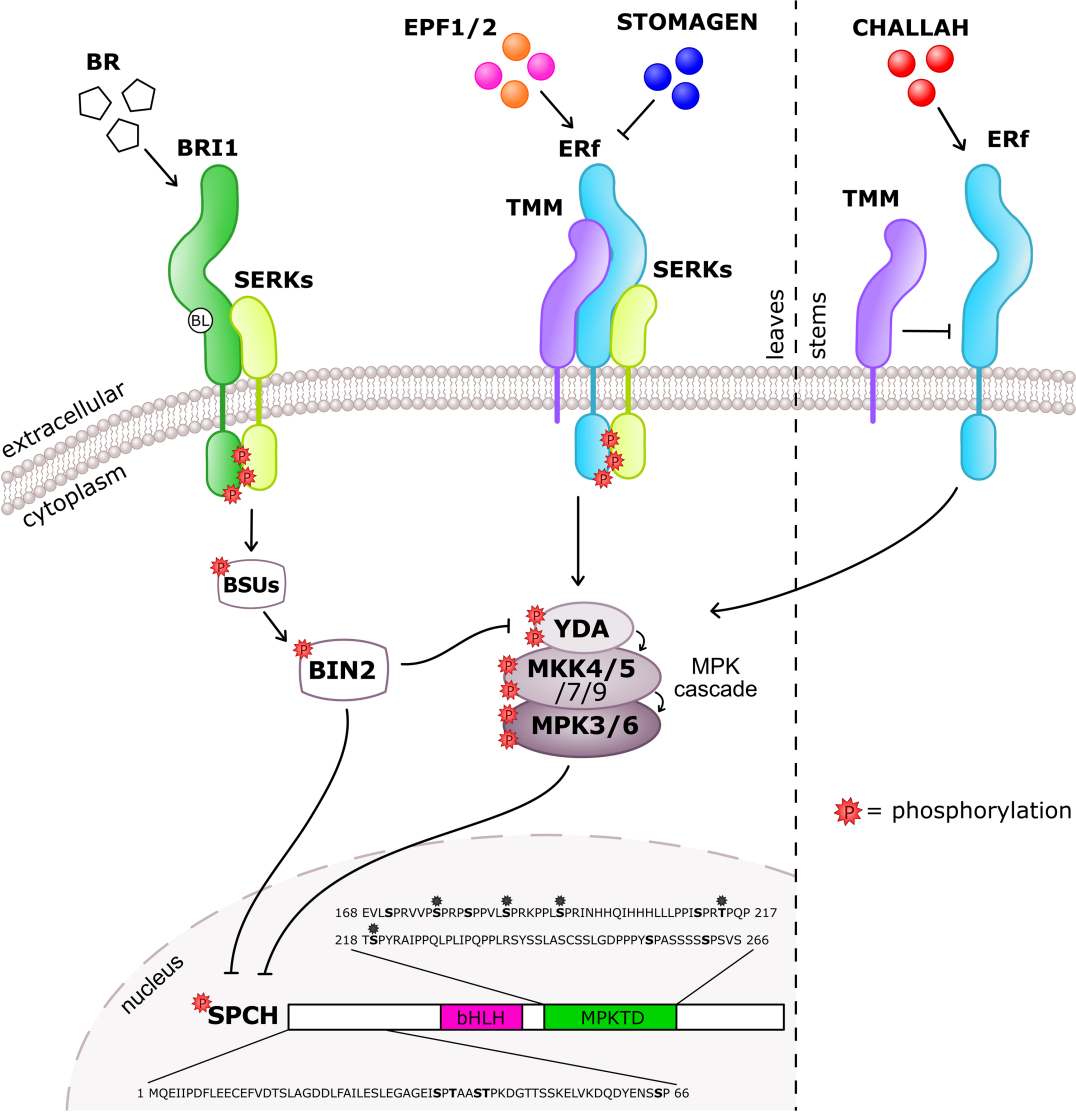
Receptor and ligand interactions govern SPCH and stomatal development. Diagram of ligand–receptor interactions that regulate SPEECHLESS (SPCH) through phosphorylation of serine and threonine residues in the N-terminus and MAPK target domain (MPKTD). The binding of EPF1/2 ligand by the ERf/TMM/SERK receptor complex activates an MPK cascade that ultimately results in the negative regulation of SPCH through phosphorylation of the MPKTD which restricts stomatal development. STOMAGEN competes with EPF1/2 for the binding sites of the Erf/TMM/SERK complex to positively regulate stomatal development. In some tissues, brassinosteroids activate BIN2 through a BRI1/SERK complex which leads to negative regulation of YDA and SPCH. In the stem/hypocotyl, TMM acts to modulate the activation of the MPK cascade by reducing the affinity of the ERf for CHALLAH. Phosphorylation sites confirmed using *in vitro* or *in vivo* techniques are shown in bold. Sites that are regulated by BIN2 and the MPK cascade are indicated by asterisks.

The differentiation of protodermal cells into stomata is regulated by three homologous basic helix-loop-helix (bHLH) transcription factors: SPEECHLESS (SPCH), MUTE and FAMA [14–16]. All three are critical to the development of stomata as mutation in any of these bHLH genes leads to the loss of properly formed stomata on the epidermal surface. The first transition into the stomatal lineage is controlled by SPCH, which promotes differentiation of protodermal cells into MMCs and their subsequent asymmetric division (Figure 1) [14,17,18]. Mutants that do not have a functional SPCH protein are unable to enter the stomatal lineage and instead form an epidermis consisting entirely of pavement cells. A meristemoid can either undergo several rounds of amplifying divisions, spacing divisions or progress down the lineage, depending on SPCH levels and activity. The proliferative role of SPCH in generating daughter cells with multipotent potential is often compared with animal stem cells [17,19]. SPCH levels and activity are highly regulated through a peptide signalling pathway, which acts through a mitogen-activated kinase (MPK) cascade (Figure 2) [18,20,21]. The scaffold proteins and receptor combinations, present in the different cell types of the stomatal lineage, influence the specificity and outcomes that the peptide signalling can achieve. SPCH is regulated by many factors including plant hormones [brassinosteroid (BR) and abscisic acid] and mechanical signals [22–25].

Like other bHLH transcription factors, SPCH, MUTE and FAMA are able to heterodimerise with other transcription factors through the helix-loop-helix domains. They interact with two other bHLH transcription factors called INDUCER OF CBP EXPRESSION 1/SCREAM (ICE1/SCRM) and SCREAM2 (SCRM2), which are critical for correct SPCH function and transcriptional regulation of downstream genes [26]. The SPCH transcription factor has been found to bind to 8327 regions in the *Arabidopsis* genome with 70% of the binding sites being in the promoter regions of genes [17]. Many of these genes are involved in cell division, peptide signalling and meristemoid fate regulation, which ensures correct spacing and divisions as the cells transition to GMCs. RNA-seq and ChIP-seq data suggest that SPCH is capable of forming a positive feedback loop in which it directly up-regulates its own expression, and the expression of *ICE1*, to drive the asymmetric cell divisions that establish meristemoid identity [17]. Although SPCH is needed to enter the lineage, the expression of *MUTE* and transition to GMC do not appear to be directly under its control. Overexpression of *SPCH* does not lead to all epidermal cells developing into stomata, but instead results in an epidermis full of ectopic cell divisions further indicating its role in promoting asymmetric divisions and amplifying cell divisions [13].

The transition from meristemoid to GMC is regulated by MUTE, which, when mutated, results in stomatal lineage cells arresting at the meristemoid cell type [15]. The lineage still undergoes amplifying and spacing divisions, indicating that MUTE is not required for initial spacing and patterning and that these processes are under the control of SPCH. Interestingly, when a constitutively active promoter is used to drive MUTE expression in the *spch* background, it is able to partially rescue the *spch* phenotype [27]. The stomata formed in the overexpression background are morphologically normal, but reduced in number. When MUTE is overexpressed in wild-type *Arabidopsis*, it creates an epidermis that is almost solely composed of stomata [15]. Overall, this suggests that while SPCH primes the epidermis with the correct spacing and patterning of meristemoids, it is MUTE which ultimately drives cells through the lineage to become stomata.

The final step of the stomata lineage is the symmetric division into the two cells that ultimately form the guard cells. This final cell division is regulated by FAMA, which simultaneously must promote guard cell identity and irreversibly terminate the meristematic activity of the lineage cells [16,28–30]. Leaves lacking FAMA are unable to produce stomata, but instead produce *fama* tumours, through a series of uncontrolled symmetrical divisions of GMCs. If FAMA expression or the canonical LxCxE domain within FAMA is altered, then FAMA is unable to correctly interact with the cell cycle regulator RETROBLASTOMA-RELATED (RBR) protein. This FAMA–RBR interaction is required in order to recruit the POLYCOMB REPRESSOR COMPLEX to switch off *SPCH* and *MUTE* expression through chromatin methylation (H3K27 trimethylation) [28–30]. Loss of correct FAMA expression or LxCxE results in the development of the unusual phenotype known as stoma-in-stoma, where guard cells have *SPCH* switched on after differentiation allowing for cell division and re-entry into the stomatal lineage. FAMA also directly activates genes needed for guard cell function, such as *POTASSIUM CHANNEL IN ARABIDOPSIS THALIANA 1* that is directly involved in stomatal aperture control [30].

## Peptide signals

As discussed above, fate decisions within the stomatal lineage are tightly controlled. One of the most well-understood regulators of stomatal development is the peptide signalling pathway. These peptides act through leucine-rich repeat receptor kinases (LRR-RKs) and associated scaffold proteins to activate an MPK cascade leading to phosphorylation of SPCH (and potentially MUTE) attenuating its activity and stability [13,18,31–35]. There is further evidence that this pathway acts at all steps in the stomatal lineage and can either negatively or positively regulate fate decisions [18,21–23,35]. In this section, we review the structure and function of the peptide signals and associated LRR-RKs that, together with the bHLH factors outlined above, regulate progression through the stomatal lineage.

Stomatal peptide signalling is underpinned by three main peptides in *Arabidopsis* leaves: EPIDERMAL PATTERNING FACTORS 1 and 2 (EPF1, EPF2) and STOMAGEN (STOM, also referred to as EPIDERMAL PATTERNING FACTOR LIKE 9, EPFL9) [32,36–39]. These peptides play agonistic roles with EPF1/2 negatively regulating stomatal development by acting as ligands to activate the LRR-RKs, while STOM is a positive regulator that is able to compete for binding of the LRR-RKs with the EPF1/2 [40–42]. All three of the peptides are expressed as propeptides that must be first cleaved by proteases in order to become fully activated. Currently, there are two subtilisin-like proteases that are known to be involved in the control of stomatal development, STOMATAL DENSITY AND DISTRIBUTION1 (SDD1) and CO_2_ RESPONSE SECRETED PROTEASE (CRSP) [43–45]. However, while CRSP has been shown to cleave EPF2, the effect that this cleavage has on the EPF2 LRR-RK interactions is unknown [44]. Recent evidence has shown that the cleavage of EPF2 by CRSP removes several amino acids involved in the EPF1 ERL1/TMM (too many mouths) and EPFL4 ERL2 interactions [41,44]. Genetic evidence suggests that cleavage by SDD1 is not required for correct function of EPF2. This analysis is complicated by potential functional redundancy within this peptide family, and currently, there is not enough evidence to determine whether SDD1 processes EPF family members or does not [36]. The functions of EPF1 and EPF2 are similar [46], but EPF2 acts slightly earlier in stomatal development and predominantly regulates SPCH and therefore meristemoid behaviour, whereas EPF1 enforces the one cell spacing rule in meristemoids, and has been shown to play a role in autocrine regulation of GMCs and in inhibiting SLGCs from entering the lineage [47]. Although both EPF1 and EPF2 affect SPCH protein levels [46], molecular and genetic evidence suggests that their functions do not entirely overlap [32,48]. Loss of EPF1 leads to breaking of the one cell spacing rule and stomatal clustering (two or more stomata touching). In contrast with EPF1/2, which are expressed by stomatal lineage cells, STOM is expressed in internal mesophyll cells during leaf development before being secreted into the apoplast [36–39,49]. Overexpression of EPF1 or EPF2 leads to a decrease in stomatal density, whereas the overexpression of STOM drastically increases stomatal density and causes clusters of stomata to form. Interestingly, *epf2* mutants do not have an increased stomatal index (percentage of stomata in total cell number per unit area), but instead have a decreased index through increased cell division, although their overall stomatal density is increased [32,36,38,39]. This increase in cell division is similar to the *SPCH* overexpression phenotype, which demonstrates that the critical role EPF2 plays in the regulation of SPCH activity.

The *Arabidopsis* EPF family are plant peptide hormones, composed of 11 secreted defensin-like cysteine-rich peptides that are able to interact with transmembrane LRR-RKs [50]. Unlike the defensin peptides, the EPF family does not have CSαβ or γ-core motifs [49–51]. Instead, the EPF family generally consists of two antiparallel β-strands with six conserved cysteines, which form three disulphide bonds. Synthesised STOM peptides that are missing even one of the conserved cysteine residues are no longer able to function correctly to increase stomatal development [40]. Although all members of the family share structural homology, in between the fourth and fifth conserved cysteines of the EPFs, there is a variable loop region that was thought to specify the antagonist actions of STOM on EPF1 and EPF2 activity. Ohki et al. [40] investigated the structure and function of STOM and EPF2 using a combination of NMR and semi *in vitro* techniques. The variable loop region of EPF2 contains two extra cysteine residues that, through using a combination of enzymatic digestion and mass spectroscopy, were found to form an extra disulphide bond [40]. Further structural analyses were carried out to investigate whether STOM antagonises EPF2 activity through direct binding to EPF2 or through competing for the EPF2 receptor [40]. NMR revealed no changes in either peptide's structure when in each other's presence. This confirmed that the antagonistic effect of STOM on EPF2 is likely due to competition for receptor binding rather than a direct interaction between these two peptides. A recent study has added further insights into the underlying mechanism of this association by using *in vitro* competition assays to show that STOM competes with EPF1 and EPF2 for binding of the LRR-RKs and their associated proteins [40–42].

Further research into the structure–function relationships of STOM and EPF2, using semi *in vitro* assays, revealed that the loop structure provides specificity to the antagonistic actions of the peptides. The creation of two chimeric peptides, one with the EPF2 peptide scaffold and the STOM extended loop and the reciprocal with a STOM peptide scaffold and the EPF2 loop, was found to mimic the activity of the respective loop structures rather than the scaffold [40]. Neither of the chimeric peptides were as potent as the wild-type scaffold-loop combinations. However, the chimeric peptides still functioned in a concentration-dependent manner, and taken together, these data clearly demonstrate that the different loop structures of STOM and EPF2 confer the function of the peptides as positive and negative regulators of stomatal development, respectively. Furthermore, NMR results indicated that the extended loop of the STOM peptide is relatively hydrophobic and the introduction of a glutamic acid-to-alanine mutation (E28A) significantly affected the ability of the peptide to enhance stomatal development. It is suggested that the reduced hydrophobicity of the mutant E28A loop renders the peptide less able to interact with the hydrophobic outer membrane domains of the appropriate LRR-RKs [40].

## Receptor signalling: the Erecta family of LRR-RKs

To co-ordinate cell division, expansion and differentiation in the developing leaf, cell-to-cell communication is required. In the *Arabidopsis* epidermis, one of the most prominent examples of this is the ‘one cell spacing rule’ of stomatal development [37]. In the peptide signals section of this review, the peptide ligands that act to positively or negatively regulate stomatal development were outlined. The current section focuses on our present understanding of the signalling cascade triggered by EPF peptide binding, starting with the LRR-RKs and finishing with the phosphorylation of the stomatal bHLHs by an MPK cascade. Plant genomes encode large families of LRR-RKs and their associated LRR receptor-like proteins (LRR-RLPs). LRR-RKs are the largest subfamily of transmembrane receptor-like kinases in *Arabidopsis* with more than 200 members [52,53]. The LRR-RKs are involved in a range of plant developmental processes from cell division and stem cell niche maintenance to defence and wounding responses. They have well-established roles as cell surface receptor complexes with the LRR domain constituting the extracellular component. Typically, LRR-RKs are the signal transducers within a pathway, activating downstream signalling cascades in response to ligand binding [52,53]. In contrast, the associated LRR-RLPs modulate the function of the LRR-RKs giving them cellular and tissue specificity. Although they share similar structural homology of extracellular LRR domains and cytoplasmic kinase domains, the LRR-RKs have a diverse range of ligands, ranging from steroid hormones (brassinosteroids) to plant-derived peptides and secreted proteins (EPF family and the CLAVATA family) to bacterial proteins (FLAGELLIN 22) [54,55].

Some of the most widely studied LRR-RKs are the ERECTA (ER) family consisting of *ER* and its two closely related paralogues *ERECTA-Like* 1 (*ERL1*) and *ERECTA-Like 2* (*ERL2*) [34]. Although they have a major role in stomatal development, the ER family regulates many other developmental processes including aspects of leaf, floral, root and vascular development. The *er* mutant was first isolated in the 1950s in the Landsberg ecotype (known as *Ler*) and soon became a model laboratory plant due to its compact rosette and inflorescence. Despite the popularity of *Ler* with geneticists, the underlying genetic mutation was not characterised until the mid-1990s [31]. Plants defective in ER function have increased stomatal density, as well as an increased number of arrested SLGCs that are unable to progress through the stomatal lineage. In some instances, the *er* mutant has been shown to have a reduced stomatal index associated with excessive cell divisions, reminiscent of the phenotype of plants lacking EPF2 [33]. However, this appears to be inconsistent across the literature with other studies indicating an increase or no change in stomatal index [33,44,56,57]. The difference in developmental signalling between leaf and cotyledon development, and SLGC regulation in these different organs may explain some of these reported discrepancies.

In comparison with *er*, the single *erl1* and *erl2* null mutants and the *erl1 erl2* double mutant exhibit less severe stomatal development phenotypes, although the number of SLGCs is reduced [33]. This indicates that ER is the more dominant member of this receptor family in regard to stomatal development. In contrast with the single mutants, the *er erl1* and *er erl2* double mutants have enhanced effects on stomatal development. The *er erl1* double mutant has an increased stomatal density and index, with the elevated numbers of SLGC seen in the *er* single-mutant proceeding through the lineage and forming stomata. The *er erl2* double mutant has a slight enhancement in SLGC number compared with the *er* single mutant [33]. Overall, this genetic analysis suggests that ERL1, together with ER, may target the GMC stage of development and the regulation of MUTE, as evidenced by the increase in stomatal density and index in the *er erl1* double mutant. MUTE directly regulates *ERL1* expression, as determined by ChIP-qPCR, and enables a cell-specific mechanism to regulate MUTE activity [47]. ERL2 signalling possibly plays a greater role in the regulation of SPCH as the *er erl2* double mutant is more phenotypically similar to *epf2* mutants or plants overexpressing *SPCH*. A mutagenesis screen recently identified VAP-RELATED SUPPRESSORS OF TMM (VST) as regulators of ER-mediated signalling [58]. The VST family acts at the plasma membrane to bind integral endoplasmic reticulum proteins, which influence signalling by modifying plasma membrane microdomains [58]. The *vst1;vst2;vst3* triple mutant phenotypically has clustered stomata and excessive cell divisions. ERL2 appears to have a unique role in the interactions with VSTs, and co-immunoprecipitation assays indicate a protein–protein interaction with ERL2 and VST1 [58]. Levels of ERL2 are also elevated in the *vst1;vst2;vst3* triple mutant, and as a direct target of SPCH, this may suggest that SPCH is overactive in the *vst1*;*vst2*;*vst3* triple mutant [17,58]. This overactivity could be compounded by ERL2 being a weak repressor of SPCH and the heteromerisation of the extra ERL2 with ER and ERL1 may inhibit strong signalling [58]. This model of ERL2 VST-family interactions does not take into account ERL1 also a target of SPCH and how this feeds into the ERf LRR-RLK dynamics [17,58]. In the *er erl1 erl2* triple ER-family mutant, the complete loss of signalling results in a breaking of the one cell spacing rule as well as an increase in stomatal density and index. As stomatal clustering is commonly observed only in the *er erl1 erl2* triple mutant, this indicates that the one cell spacing rule only requires one functional ER-family member [33,34]. Lee et al. [42] tested the ability of STOM to signal through the various ER-family receptors and found that STOM induction altered stomatal development in both single and double ER-family mutants. However, STOM induction had no effect in the triple *er erl1 erl2* mutant or in mutants in the LRR-RLP family, *TMM*, indicating that TMM and at least one ER-family LRR-RLK are required for the peptide signal to enhance stomal development [42].

## TMM co-receptor

LRR-RKs often form working complexes with other LRR-RKs and LRR-RLPs; the different combinations of these LRR-RKs/LRR-RLPs complexes allow for cell-type-specific signalling to be achieved. In stomatal development, there is one key LRR-RLP, TMM, which is critical to the correct spacing and patterning of stomata within the epidermis [13]. TMM was one of the first stomatal regulators characterised and, as the name suggests, *tmm* mutants produce an excess number of stomata that form in large clusters. Although TMM lacks a cytoplasmic kinase domain, it has a crucial role in forming the active extracellular complexes that are necessary to perceive peptide signalling. TMM is often used as a marker for the stomatal lineage as it is only expressed in stomatal precursor cells and young guard cells. In fact, *TMM* is a direct target of the SPCH transcription regulator and is part of an autoregulatory mechanism whereby SPCH limits the number of cells that enter the lineage and the number of amplifying divisions that occur [17,59]. In contrast with the leaves, which have clustered stomata, the stem and hypocotyl of *tmm* mutants have no stomata [13]. This curious juxtaposition of the two different *tmm* phenotypes was not resolved until recently when Lin et al. [41] showed that the tissue-specific expression of the LRR-RKs in conjunction with TMM is able to create precise grooves to interact with the different EPF members [41].

In the leaf epidermis, TMM binds to ER and ERL1 to create a specific plasma membrane receptor complex for EPF1 and EPF2 [48]. This has been demonstrated by co-crystallisation of the LRR domains of ERL1 and TMM in conjunction with peptide-binding assays [41]. Co-expression of TMM with either ER or ERL1 formed molecular complexes with ∼1 : 1 stoichiometry, and co-crystallisation of the TMM and ERL1 LRR domains exposed some of the molecular mechanisms behind this interaction. The concave surface of TMM contains a neutral charge in the centre, which leads to the establishment of van der Waals interactions with the convex surface of ERL1 [41]. In contrast, the periphery of the concave surface of TMM is positively charged and this complements the N-terminal side of ERL1. Using site-directed mutagenesis, Lin et al. [41] confirmed interacting residues identified in the ERL1–TMM crystal structure as essential for the formation of the ERL1–TMM complex. Mutating key residues in both TMM and ERL1 have been shown to disrupt complex formation *in vitro*. However, when tested *in vivo*, these single amino acid mutants of ERL1 and TMM were still able to complement their respective mutant backgrounds, suggesting that their close proximity in the plasma membrane is able to overcome the weaker interactions or that other factors stabilise their interaction [41]. Binding assays confirmed that this deep interaction between TMM and ERL1 is necessary for the perception of the EPF1 and EPF2 peptides in the leaves. When expressed without TMM present, the ER family was unable to bind EPF1 or EPF2, which confirms that the *tmm* phenotype of clustered stomata is due to the developing epidermis being unable to perceive peptide signals. The binding of EPF1 by the TMM–ERL1 complex did not induce dimerisation of ERL1, which is in agreement with the need for co-receptors to activate downstream signalling [41,60].

Lin et al. [41] further examined the ability of the ER family to bind other members of the EPF family revealing different interactions to those previously reported. *CHALLAH* (*EPFL4*) is primarily expressed in developing hypocotyl, inflorescent meristem and stem tissue in *Arabidopsis* where it can act as a negative regulator of stomatal development when overexpressed [61,62]. In *tmm* mutants, the hypocotyl and stem tissues lack stomata and genetic evidence suggested that this is due to CHALLAH acting as a strong suppresser of stomatal development when *tmm* is missing [61,62]. Binding assays revealed that in contrast with EPF1 and EPF2, the CHALLAH-related members of the EPF family (EPFL4, 5, 6) were found to be able to interact with the ER-family LRR-RLKs without TMM [41,63]. This indicates that TMM normally acts to modulate EPFL peptide interaction with the ER family in the stem and prevents constant activation of the MAPK pathway and the subsequent loss of stomatal development. Modulation by TMM also explains why overexpression of CHALLAH in wild-type backgrounds has a minimal impact, whereas overexpression of CHALLAH in *tmm* can partially rescue its phenotype [61]. Interestingly, crystallisation of the ERL2–EPFL4 complex revealed that it was similar in structure to the ERL1–EPF1 complex, suggesting that although TMM is needed for ERL1–EPF1 recognition, ERL1–EPF1 and ERL2–EPFL4 may share downstream co-receptors [60]. Overall, the genetic and biochemical evidence suggests that the evolution of TMM and its specific role in regulating stomatal development is critical for correct peptide recognition by the ER family (Figure 2).

## Somatic embryogenesis receptor kinase

The intricate nature of the different peptide ligands and their receptor complexes has been discussed in depth; however, a seemingly unrelated LRR-RK family has been found to be necessary to couple extracellular ligand recognition to intracellular signalling. This family is known as the *SOMATIC EMBRYOGENESIS RECEPTOR KINASEs* (*SERKs*) and has been previously implicated in stomatal development, plant immunity, programmed cell-death regulation and BR signalling [60,64–66]. SERKs are an ancient family of LRR-RKs present in several algae species and in liverworts [64]. In *Arabidopsis*, there are five homologues (*SERK1* to *SERK5*), and they are characterised by the presence of a serine–proline-rich region in the extracellular domain [64]. Despite their homology, SERKs have been recruited to perform differential functions in a complex code; *SERK3* (also called *BAK1*) and *SERK4* are involved in regulating programmed cell death and immunity [65,66]. *SERK1*, *SERK3* and *SERK4*, but not *SERK2*, are essential in BR signalling. No phenotype has been associated with *SERK5*, which has led to the hypothesis that it is a pseudogene. Using genetic analysis, Meng et al. [60] revealed that, in descending order, *SERK3*, *SERK2*, *SERK1* and *SERK4* have important but redundant roles as co-receptors for peptide signalling in stomatal development. Individual null mutants of each SERK do not affect stomatal development [60], nor do higher-order mutants with the exception of the *serk1-1/serk2-1/bak1-4* triple mutant, which shows a high level of stomatal clustering. Interestingly, no other triple mutant combination presented with a stomatal phenotype, suggesting redundancy between *SERK1*, *SERK2* and *SERK3*. To assess a quadruple *serk* mutant, *bak1-5* semi-dominant allele was used to circumvent embryo lethality. This *serk* quadruple mutant showed a similar level of clustering and stomatal index as the *er erl1 erl2* triple mutant, and double and triple mutants containing the *bak1-5* allele all showed increased stomatal indexes and stomatal clustering of varying levels of severity [60]. Although BR is known to affect stomatal development through the phosphorylation of SPCH, *bak1-5* double and triple mutants all display a wild-type response to BR, suggesting that their stomatal phenotype is independent of BR signalling [60].

Since the role of the SERK family in stomatal regulation is most probably independent of BR signalling, their ability to interact with the ER family and TMM was further investigated. Using co-immunoprecipitation assays, the SERKs were found to interact with TMM and ER-family members, with SERK3 being able to interact with ER and ERL1 when expressed from their native promoters. The application of bioactive EPF1 and EPF2 peptides induced a stronger association between the SERKs, ER and ERL1 [60]. *In vitro* kinase assays suggest that SERK3 and ER are able to phosphorylate each other's cytosolic kinase domains, potentially activating downstream signalling through transphosphorylation [60]. Taken together, this suggests that the SERKs form complexes with TMM and the ER-family members that are essential for eliciting intracellular signalling through the MPK cascade (Figure 2). SERK3 crystallisation with LRR-RLKs, BL-BRI1 and flg22-FLS2 has shown that SERK3 does not confer ligand-binding activity, but interacts with the ligand receptor complexes directly to elicit a response [67,68]. However, another LRR-RLK receptor, HAESA, has been shown to be SERK dependent in binding the peptide ligand IDA [69]. More investigation into the interactions of SERKs/ERf/TMM/EPFs will clarify the exact role of SERKs in stomatal development.

## Mitogen-activated kinase cascade

Once the cytosolic kinase domains of the ER/SERK/TMM/EPF complexes become phosphorylated, it triggers a downstream series of phosphorylation events [18,21,35,70]. This ultimately results in a change in phosphorylation state in one of the three master regulators of stomatal development. MPK cascades have been shown through biochemical and genetic evidence to connect extracellular signalling to intracellular repression of the stomatal lineage [18,20]. The target of ER/SERK cytosolic kinase domains is a mitogen-activated protein kinase kinase kinase (MPKKK) named YODA (YDA). *yda* mutant plants produce excessive and clustered stomata, but are often embryo lethal and defective in several other developmental and physiological responses that utilise the MPK cascade [21]. Genetic analysis of double mutants supports YDA's role as a downstream regulator of stomatal development; ΔN-YDA, a constitutively active form, confers an opposite phenotype to *yda* and is able to rescue the phenotype of both *tmm* and *sdd1* [21]. The phenotype conferred by ΔN-YDA is similar to that of a *spch* mutant and produces leaves with no stomata and an epidermis consisting entirely of pavement cells.

YDA, in turn, phosphorylates the next class of proteins in the MPK cascade, the mitogen-activated protein kinase kinases (MPKKs). In *Arabidopsis*, there are 20 *MPKKs*, although only four (*MPKK4/5/7/9*) are implicated in stomatal development. Using cell-type-specific expression, Lampard et al. [70] showed that MPKK4 and MPKK5 are strong negative regulators of stomatal development and can inhibit protodermal cells from progressing through the lineage [35,70]. All four MPKKs implicated in stomatal development are able to repress the transition of meristemoid to GMC. However, when MPKK7 and MPKK9 are constitutively activated in GMC cells, they cause the overproduction and clustering of stomata, indicating that cell-type specificity regulates MPK signalling in the stomatal lineage [35,70].

The final step in the MPK cascade is the phosphorylation of mitogen-activated protein kinase (MPKs) by the MPKKs. There are at least 20 *MPKs* in *Arabidopsis*, although only three (*MPK3/4/6*) have been studied and implicated in a wide range of stress and developmental processes. Unlike earlier steps in the phosphorylation cascade, MPKs directly interact with and phosphorylate the targets of the MPK cascade. MPK phosphorylation of transcription factors can lead to a wide range of effects that bring about activation, degradation or conformational changes resulting in new protein–protein interactions. *In vitro* co-immunoprecipitation assays have revealed direct interactions between MPK6/MPK3 and SPCH, but not MUTE or FAMA. MPK4 has been shown to target MUTE for phosphorylation *in vitro*; however, there remains to be any *in vivo* or phenotypic evidence that the phosphorylation of MUTE is regulatory [18,20,35]. This lack of MUTE phosphorylation is perplexing, as MUTE directly activates ERL1 transcription to perceive extracellular regulatory signals. Deletion of the MAPK target domain (MPKTD) from SPCH stops the interaction with MPK3 and MPK6, and when the truncated SPCH is expressed in plants, it causes the formation of stomatal clustering. At present, genetic and biochemical evidence has shown that stomatal development is regulated by the MPKKK (YDA), cell-type-specific MPKKs (MPKK4/5/7/9) and MPK3/6. This biochemical system allows for developmental flexibility in stomatal development. If more STOM is present than EPF2 in the extracellular space outside the SLGC, it will out-compete for receptor binding and prevent activation of the stomatal MPK cascade. SPCH levels will be maintained and the SLGC will divide to form another meristemoid and SLGC pair.

MPK signalling cascades are fundamental for many developmental, stress and physiological responses. However, how they activate specific downstream targets remains largely unknown. One potential explanation is that individual cell types express scaffold proteins, which associate with MPK cascade components to add specificity. In the stomatal lineage, one such protein, BREAKING OF ASYMMETRY IN THE STOMATAL LINEAGE (BASL) [71], has been implicated as a scaffold for the stomatal MPK cascade acting to correctly regulate asymmetric cell divisions [71–73]. In *basl* mutants, stomata are paired, thus breaking the one cell spacing rule [71]. In *Arabidopsis*, following the asymmetric cell division of an MMC to produce an SLGC and a meristemoid, SPCH is normally maintained in the meristemoid but degraded in the larger SLGC by the activity of the stomatal MPK cascade [72,73]. In contrast, loss of BASL results in defective asymmetric divisions with both daughter cells progressing through the lineage to form stomata. BASL is normally asymmetrically distributed before the asymmetric division, with the larger SLGC inheriting more of the scaffold at the plasma membrane and therefore more of the MPK cascade. BASL itself is phosphorylated by MPK3/6, and this helps to maintain MPK cascade activity and BASL localisation [72,73]. Although both cells inherit SPCH equally from the meristemoid mother, the large amount of BASL and MPK cascade activity rapidly degrades the SPCH pool in the larger daughter cell. Exactly, how BASL is able to target MPK6 to be able to phosphorylate the nuclear-located SPCH and enhance its degradation, however, remains an unanswered question.

## Regulation of SPEECHLESS by phosphorylation

If left unchecked, master regulators of developmental processes could cause reduced fitness or lethality. SPCH, MUTE and FAMA are subject to constant and precise regulation in their control of the formation and patterning of the epidermis. Peptide signalling acting through LRR-RKs and the stomatal MPK cascade function to phosphorylate SPCH, to reduce its concentration and regulate its activity. The MPKTD in SPCH is a major regulatory region for MPK phosphorylation that is not found in either MUTE or FAMA. Using site-directed mutagenesis, the role of several potential phosphorylation sites in controlling SPCH's role in asymmetric cell division and GMC fate promotion has been investigated (Figure 2). Differential phosphorylation of the SPCH MPKTD has been shown to influence its behaviour [17,18,35,74]. SPCH variants that have all five phosphorylation sites mutated to alanine (SPCH1-5A) show a greater ability to promote cells to GMCs. In contrast, when only the first four phosphorylation sites are mutated to alanine (SPCH1-4A), SPCH activity is restricted to driving asymmetric division [74]. Further investigation revealed that SPCH1-5A is able to complement the *mute* null allele when driven by a strong inducible promoter or the native *MUTE* promoter. Indeed, deletion of the entire SPCH MPKTD results in clustered stomata, a phenotype normally associated with increased MUTE activity. The presence of just a single phosphorylation site changes SPCH's behaviour into driving asymmetric cell division [74]. Taken together, this suggests that during evolution, the divergence in cellular function of SPCH and MUTE was through the gain of phosphorylation and loss of phosphorylation sites, respectively.

Although MPK phosphorylation of SPCH is the most extensively researched, other signalling pathways also regulate SPCH function through phosphorylation. Like peptide signalling, BR signalling works through a complex of LRR-RKs and co-receptors to phosphorylate BRASSINOSTEROID INSENSITIVE 2 (BIN2) [22,23,60]. Phosphorylated BIN2 appears able to both phosphorylate and negatively regulate SPCH, or indirectly activate SPCH through the phosphorylation and negative regulation of YDA. The treatment of plants with BR has a stabilising effect on SPCH as seen through immunoblotting, although no increase in *SPCH* transcript levels is detected [22]. The stabilising effect could be through BIN2 being able to relieve the inhibition of YDA on SPCH (Figure 2). Differences in growth conditions and tissue types between studies have increased the complexity of interpreting the genetic analyses, as *bin2* stomatal phenotypes appear to be rescued under certain growth conditions [22,23].

Unlike the phosphorylation events downstream of MPK and BR signalling, CYCLIN-DEPENDANT KINASE A;1 (CDKA;1) phosphorylation of SPCH at serine 186 leads to an increase in stomatal density, suggesting that this modification stabilises SPCH [75]. In support of this, SPCH SER186 to ALA phosphorylation mutants are unable to complement the *spch* null allele, indicating that phosphorylation of SER186 is necessary for SPCH activity. Further support for this comes from *cdka;1* mutants which have undetectable levels of SPCH protein and produce no stomata in their epidermis [75]. Overall, SPCH has complex and vast network of post-translational modifications to control its behaviour. As SPCH helps regulate entry into the stomatal lineage and the cell number of the epidermis, this complex network is necessary to control SPCH and modulate its differential functions.

## Concluding remarks

The ability of plants to alter their developmental programmes enables them to respond to changing environmental conditions and is critical for their success. A well-studied aspect of such developmental control is that of stomatal development. Here, as in other emerging plant development pathways, regulation comes from the complex interaction of LRR-RKs, LRR-RLPs and secreted peptide ligands. The antagonistic relationship of EPF1/2 and STOM peptides carefully balances the phosphorylation status and activity of the transcription factor SPCH to ensure that the distribution of cells produced in the epidermis is appropriate for the prevailing environmental conditions. However, precisely how environmental conditions mechanistically interact with SPCH and its many regulators are largely still to be answered. Recent biochemical data, revealing the extracellular interactions between the EPF family ligands and the TMM ER family receptor complexes, have clarified how stomatal fate is initiated, and how different tissues and cell types can elicit different developmental responses. It is clear that downstream of TMM ER-family receptor activation, the signal is transduced by an MPK cascade, which along with other kinases can phosphorylate and inactivate (or activate) the SPCH transcription factor which promotes entry into the stomatal lineage. However, MPK3 and/or MPK6 are key components of many plant signalling pathways, and so, how specificity to target SPCH is informed following their activation is still to be understood. Despite these outstanding questions, it is clear that the regulation of stomatal development occurs through a complex set of post-translational modifications and protein interactions triggered by carefully balanced opposite forces that adjust gas exchange and enhance overall plant fitness. Nonetheless, the majority of stomatal development research has so far focused on the eudicot *Arabidopsis* and much less is known about other species. Remarkably though, despite the notable differences between the stomata of monocotyledonous and dicotyledonous plants, it is becoming apparent that several components of their stomatal developmental pathways, including the peptide ligands and transcription factors, are similar [76,77]. Further research will be necessary to understand how well these regulatory pathways have been conserved throughout the plant kingdom.

## Abbreviations

BASL: BREAKING OF ASYMMETRY IN THE STOMATAL LINEAGE
bHLH: basic helix-loop-helix
BIN2: BRASSINOSTEROID INSENSITIVE 2
BR: brassinosteroids
CRSP: CO_2_ RESPONSE SECRETED PROTEASE
EPF1, EPF2: EPIDERMAL PATTERNING FACTORS 1 and 2
ER: ERECTA
*ERL1*: *ERECTA-Like* 1
ICE1/SCRM: INDUCER OF CBP EXPRESSION 1/SCREAM
LRR-RKs: leucine-rich repeat receptor kinases
MMC: meristemoid mother cell
MPK: mitogen-activated kinase
MPKKK: mitogen-activated protein kinase kinase kinase
MPKKs: mitogen-activated protein kinase kinases
MPKs: mitogen-activated protein kinase
MPKTD: MAPK target domain
RBR: RETROBLASTOMA-RELATED
RLPs: receptor-like proteins
SDD1: STOMATAL DENSITY AND DISTRIBUTION1
*SERKs*: *SOMATIC EMBRYOGENESIS RECEPTOR KINASEs*
SLGC: stomatal lineage ground cell
STOM: STOMAGEN
TMM: too many mouths
VST: VAP-RELATED SUPPRESSORS OF TMM
YDA: YODA
